# Medicinal Plants and Natural Products Used in Cataract Management

**DOI:** 10.3389/fphar.2019.00466

**Published:** 2019-06-13

**Authors:** Devesh Tewari, Ovidiu Samoilă, Diana Gocan, Andrei Mocan, Cadmiel Moldovan, Hari Prasad Devkota, Atanas G. Atanasov, Gokhan Zengin, Javier Echeverría, Dan Vodnar, Bianca Szabo, Gianina Crişan

**Affiliations:** ^1^Department of Pharmacognosy, School of Pharmaceutical Sciences, Lovely Professional University, Phagwara, India; ^2^Department of Ophthalmology, Iuliu Haţieganu University of Medicine and Pharmacy, Cluj-Napoca, Romania; ^3^Department of Pharmaceutical Botany, Iuliu Haţieganu University of Medicine and Pharmacy, Cluj-Napoca, Romania; ^4^Graduate School of Pharmaceutical Sciences, Kumamoto University, Kumamoto, Japan; ^5^Institute of Genetics and Animal Breeding, Polish Academy of Sciences, Jastrzębiec, Poland; ^6^Department of Pharmacognosy, University of Vienna, Vienna, Austria; ^7^Department of Biology, Faculty of Science, Selcuk University, Konya, Turkey; ^8^Departamento de Ciencias del Ambiente, Facultad de Química y Biología, Universidad de Santiago de Chile, Santiago, Chile; ^9^Department of Food Science, University of Agricultural Sciences and Veterinary Medicine of Cluj-Napoca, Cluj-Napoca, Romania; ^10^Department of Anatomy, Iuliu Haţieganu University of Medicine and Pharmacy, Cluj-Napoca, Romania

**Keywords:** medicinal plants, natural products, cataract, antioxidant, aldose reductase, lens opacity, MAPK

## Abstract

Cataract is the leading reason of blindness worldwide and is defined by the presence of any lens opacities or loss of transparency. The most common symptoms of cataract are impaired vision, decreased contrast sensitivity, color disturbance, and glare. Oxidative stress is among the main mechanisms involved in the development of age-related cataract. Surgery through phacoemulsification and intraocular lens implantation is the most effective method for cataract treatment, however, there are chances of serious complications and irreversible loss of vision associated with the surgery. Natural compounds consisting of antioxidant or anti-inflammatory secondary metabolites can serve as potential leads for anticataract agents. In this review, we tried to document medicinal plants and plant-based natural products used for cataract treatment worldwide, which are gathered from available ethnopharmacological/ethnobotanical data. We have extensively explored a number of recognized databases like Scifinder, PubMed, Science Direct, Google Scholar, and Scopus by using keywords and phrases such as “cataract”, “blindness”, “traditional medicine”, “ethnopharmacology”, “ethnobotany”, “herbs”, “medicinal plants”, or other relevant terms, and summarized the plants/phytoconstituents that are evaluated in different models of cataract and also tabulated 44 plants that are traditionally used in cataract in various folklore medical practices. Moreover, we also categorized the plants according to scientific studies carried out in different cataract models with their mechanisms of action.

## Cataract: An Overview

The crystalline lens lies behind the iris and represents the dynamic part of the eye’s optical system, responsible for focusing the image onto the retina. Cataract is defined by the presence of any lens opacities or loss of transparency. The most common symptoms of cataract are impaired vision, decreased contrast sensitivity, color disturbance, and glare. Changes in the lens may also serve as markers for systemic health and aging in the over-all population (Song et al., 2014). According to the type of lens opacities, cataract is classified into three classical types: nuclear, posterior subcapsular, and cortical. These types can also be associated with each other and if untreated, they progress to total lens opacification. Some of the most common causes for cataract in adults are age, diabetes, steroid use, family history, or trauma. Congenital cataract has a significant prevalence, also.

Cataract is the foremost reason of blindness worldwide in spite of the technological advancements in eye surgery in the last two decades. In 2010, there were around 32 million blind people and 191 million were with poor vision. One in three blind people suffered from cataract (Khairallah et al., 2015). The World Health Organization (WHO) suggests that by 2020 the number of blind people will reach 90 million globally (Khairallah et al., 2015; Taylor, 2016). The strategy to fight this challenge is costly, aiming human resource, infrastructure development, and effective disease control. The latter is dependent on the characteristics of the specific disease. Prevalence of cataract increases with age, from 5% for patients of age 52–62 to 64% for patients over 70 years, in Europe (Prokofyeva et al., 2013). Age is a non-modifiable risk factor involved in the pathogenesis of cataract, hence the progressive aging of the population is an alarming issue. Identifying modifiable risk factors for cataract is imperative and may help to establish the preventive measures.

The surgical treatment for cataract consists of cataractous lens extraction and intraocular lens implant. It is the only current treatment available in order for patients to recover their visual function. This implies a significant cost and there is a significant lack of access to surgery, especially in the developing world. Despite good postoperative outcomes, complications are possible following cataract surgery. Studies have suggested that pseudophakia patients have a higher risk of retinal detachment. Endophthalmitis has also been reported in 0.12% of the operated cases (Toh et al., 2007). After the surgery, the mobility of the lens is lost and correcting glasses are usually necessary. This will only increase the expense and the discomfort for the patient and society. Medical treatment would be a desired alternative.

The most primitive written reference to cataract surgery was discovered in Sanskrit manuscripts dating back from the 5th century BCE. It was attributed to *Sushruta*, a well-known ancient plastic surgeon who described a procedure known as couching, in which the cataractous lens was displaced with a sharp tool to fall it into the vitreous cavity, clearing the visual axis, though the vision was significantly blurred as there were no corrective lenses or glasses (Uhr, 2003; Sachdev, 2017). Even at the time of Mesopotamia (ca. 3,000–4,000 BCE) records reveal that mysticism along with different animal products, vegetables, and minerals were utilized for the treatment of devil and spirits causing eye diseases. Hundreds of remedies were also described during the Greek era (ca. 460–375 BCE) for disorders of the eyes. Moreover, eye diseases are also described anatomically by *Sushruta* (as mentioned above), Galen and various medicinal and surgical procedures were described for the treatment of eye diseases (Duke-Elder, 1962; Albert and Edwards, 1996; Goodman, 1996). In 1748, the introduction of modern cataract surgery was done by Jacques Daviel in Paris, in which the cataractous lens is removed from the eye. Later on in 1753, Samuel Sharp of London presented the intracapsular procedure, wherein the whole lens was removed by an incision by put on thumb pressure. In 1867 silk sutures for cataract surgery was originally described by Henry Willard Williams of Boston (Uhr, 2003).

## Cataract – Pathogenesis

Various mechanisms have been associated with age-related cataract pathogenesis. Lens opacities may appear due to changes in the microarchitecture, caused by mutations, biomechanical, or physical changes.

### Mutations

Despite cataract being a multifactorial disease, sometimes mutations alone can cause lens opacities and this usually leads to congenital or pediatric cataract. Studies have presented more and more evidence that genetic factors are also part of age related cataract pathogenesis, raising the probability of molecular genetic relations between lens development and aging (Hejtmancik and Kantorow, 2004). Out of around 42 genes and loci that have been found to underlie congenital forms of cataract, a few of them have been linked with age associated cataract: EPHA2 (encodes a member of ephrin receptor of protein-tyrosine-kinases), CRYAA, CRYGS (both encode lens proteins), FYCO1 (encodes a scaffolding protein which is active in microtubule transport of autophagic vesicle), or TDRD7 (encodes an RNA-binding protein). The mutation p.Gly18Val in CRYGS results in a protein with normal structure in physiological conditions. The alterations in its structure occur after thermal or chemical injury. A similar mutation is Phe71Leu in CRYAA. The discovery of mutations in genes coding for TDRD7, EPHA2, and FYCO1 has provided the initial evidence for the functional importance of posttranscriptional mRNA regulation, ephrin signaling, and the autophagy pathway, respectively, in human lens transparency (Shiels and Hejtmancik, 2015).

Gene mutations underlying secondary forms of cataract could also play part in age related cataract formation. A mutation in gene on 17q of galactokinase 1 (GALK1) which is responsible for encoding of the first enzyme in galactose metabolism, trigger autosomal recessive GALK1 1-deficiency with hypergalactosemia and cataract as a result of galactitol accumulation and osmotic stress. A coding variation in GALK1 (p.A198V) generates enzyme instability associated with amplified risk of age-related cataract in the Japanese population (Okano et al., 2001).

### Oxidative Stress

Oxidative stress is among the main mechanisms involved in the development of age-related cataract. Oxidative stress occurs when reactive compounds like the superoxide anion, hydroxyl radicals, and hydrogen peroxide are not neutralized by antioxidant enzymes and defense systems. Enzymes like catalase, SOD, and GPX are crucial for the homeostasis of the antioxidant system and ROS. When levels of ROS increase, this denatures the lens nucleic acids, proteins, and lipids, leading to mutations and cell apoptosis. Metabolic activities mostly take place in the lens epithelium. The lens epithelium uses the antioxidative enzymes in order to prevent damages caused by oxidative stress. Studies suggest that the highest concentration of SOD is in the lens epithelium (Rajkumar et al., 2013). These enzymes are also present in other parts of the lens and play a very important part in maintaining the lens clarity (Chang et al., 2013). SOD is responsible for converting superoxide anion into hydrogen peroxide, and then hydrogen peroxide is transformed into water by catalase or GPX. SOD enzyme activity is associated with cofactors like zinc, manganese, and copper. However, a decreased level of cofactors in cataractous lenses was not found. Experimental animal models show a decreased level of glutathione in the nucleus, therefore there is a higher susceptibility for oxidative damage and opacity formation (Giblin, 2000). Studies have shown that serum and aqueous humor levels of antioxidative enzymes are decreased in patients with cataract. However, there was no significant difference among different types of cataract and enzymes serum levels (Ohia et al., 2005; Wang et al., 2015).

### Crystallins Problems

Crystallins, the major structural lens proteins have an imperative role in the lens transparency and acquire post-translational alterations during cataract formation, which lead to protein insolubility, aggregation and loss of lens transparency. Out of the three major crystallins, α-, β-, and γ-, α crystallins exhibit chaperone like activity, preventing them to aggregate. The chaperone activity is reduced in cataractous lenses. Prolonged hyperglycemic conditions increase the chances of crystallins deterioration (Reddy et al., 2014). Calcium activates calcium-binding proteins triggering changes in the shape and charge of the proteins. Elevated levels of calcium appear to induce proteolysis of crystallins by calpain, an intracellular cysteine protease. Activation of calpain, an intracellular cysteine protease, leads to proteolysis of the lens proteins. In order for calpains to activate, a high level of calcium is required (Obrosova et al., 2010). Studies demonstrate that the privation of an endogenous inhibitor of calpain, named calpastatin, could be linked to the initial changes that cause cataract (Nakajima et al., 2014). Some antioxidants have been reported to regulate calcium influx in selenite induced cataracts, for instance the flavonoid fraction of *Brassica oleracea* (Vibin et al., 2010).

### Protein Structures

Alterations in the protein structure are also determined by UV exposure. Studies have shown that UVB generates more damage than UVA and that damages are prevented by the lens filters. After UV radiations, proteins suffer chemical reactions resulting in aggregations, decreasing the transparency of the lens (Cetinel et al., 2017). The crystalline lens is particularly exposed to phototoxic damage, because it absorbs most of UV radiation, together with cornea. The main association is with cortical cataract, most of the absorption occurring at the posterior surface of the lens. UV radiation can generate free radicals including oxygen-derived species, that cause lipid peroxydation of cellular membranes or can damage DNA directly (Youn et al., 2011). *In vivo*, induced cataract has no absolute threshold for UV exposure. UV induced cataract for *in vivo* exposure at UV-300 nm has a continuous dose-response function (Söderberg et al., 2016). UV radiation data from Eurosun library implied that rates of cataract were higher in regions with higher ambient UV-B radiation levels (Delcourt et al., 2014).

## Medicinal Plants and Natural Products Used Against Cataract

Opacity of the lens is triggered by free radicals in most of the cases (Varma et al., 1984; Thiagarajan and Manikandan, 2013). Severe oxidative stress also leads to the protein modifications by free radicals, and several natural products from plants are helpful in the prevention of proteins insolubilization, which may delay the opacity of lenses (Bhadada et al., 2016b). Natural compounds constituting of antioxidant or anti-inflammatory secondary metabolites could be viewed as potentially optimal anticataract agents as antioxidant effect is among the major mechanisms for prevention of cataract in most of the cases, however, not all the plants possessing antioxidant potential could have anticataract properties. The role of plant polyphenols in anti–cataractogenic activities is also studied in the comprehensive manner either *in vitro* or *in vivo* (Rooban et al., 2009, 2011; Kim et al., 2011c; Wang et al., 2011; Sasikala et al., 2013; Sunkireddy et al., 2013; Ferlemi et al., 2016).

Although there is substantial basic and applied research in the field of cataract management by natural products, mostly ethno-pharmacological/ethnobotanical research, there are not many review papers available about the activity analysis of natural products against different cataract models. One paper focused on antioxidant containing plants against cataract was found with 41 plants investigating anti-cataract activity (Thiagarajan and Manikandan, 2013). Although there are few ethnopharmacological surveys and their reviews available (Maregesi et al., 2017), there is no detailed review available on the activities of different plants extracts and natural products in cataract models.

## Methodology and Hypothesis

In this work, we attempted to gather and document the widely scattered information from various preclinical investigations and ethnopharmacological reports. We searched several web databases namely, Scifinder, ScienceDirect, Pubmed, Scopus, and Google Scholar. Boolean information retrieval method (Pohl et al., 2010) was applied using plant name with “AND” operator as also done in some other systemic reviews (Tewari et al., 2017, 2018) followed by “cataract” and using other different keywords such as “cataract”, “traditional medicine”, “ethnobotany”, “sodium selenite”, and “ethnopharmacology”.

The main research question we try to address in this paper is: “are medicinal plants/natural products used in various folk and traditional medicine of importance in the management of cataract?” and “what are the major preclinical *in vitro*/*in vivo* models that are used globally for the evaluation of cataract?”. We hypothesize that plants used in ethnomedicine are not only of potential importance but also preclinical studies conducted on various models of cataract could result in the development of potential drug candidates in future. This could be very rewarding for the scientists and scholars working in this area and also very beneficial for the patients to take forward the preclinically effective plants for clinical studies.

## Results

Oxidative stress is involved in activation of MAPKs. Compounds resulted from the activation of MAPKs have been studied and were associated with cell apoptosis. The p38 MPAK was studied *in vitro* and it was shown that it is activated by hydrogen peroxide, induce cell apoptosis in lens epithelial cells and the antioxidant agents could reduce its effects. Inhibitors of p38 MAPK reduced ROS levels and apoptosis (Bai et al., 2015).

**FIGURE 1:**
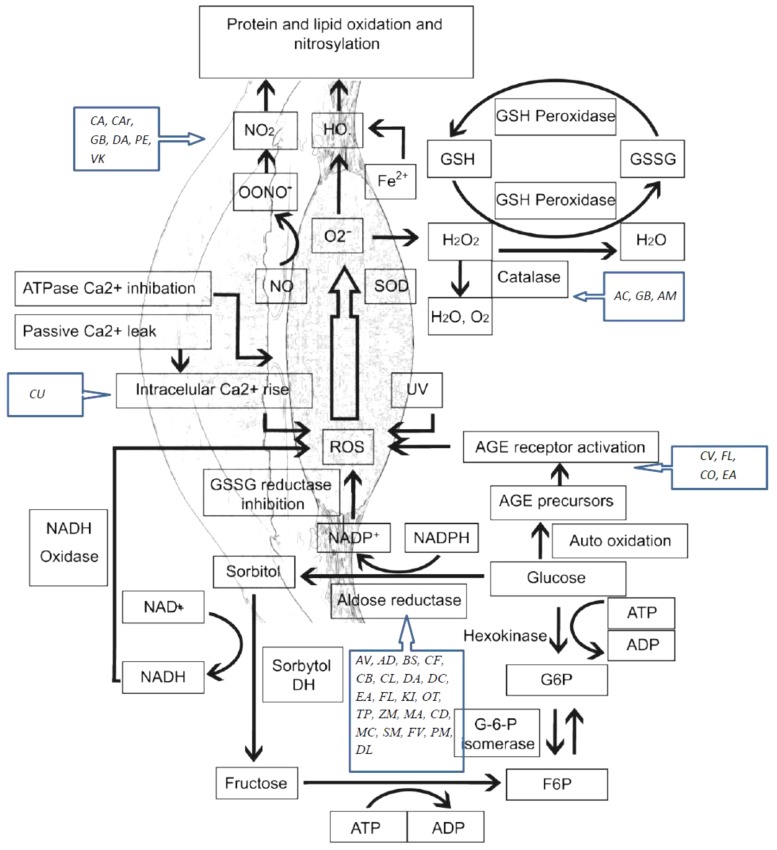
Oxidative stress mechanisms involved in cataract etiology and action mechanisms of several medicinal plants with conducted pharmacological studies for the treatment of cataract. AGE, advanced glycation end-products; ROS, reactive oxygen species; SOD, superoxide dismutase; GSH, glutathione; GSSG, glutathione disulfide; NO, nitric oxide. *AC*, *Allium cepa*; *CA*, *Coffea arabica*; *CU*, *Curcumin*; *GB*, *Ginkgo biloba*; *AV*, *Adhatoda vasica*; *AM*, *Aegle marmelos*; *AD*, *Angelica dahurica*; *BS*, *Biophytum sensitivum*; *CB*, *Caesalpinia bonduc*; *CF*, *Cassia fistula*; *CV*, *Cinnamomum verum*; *CL*, *Curcuma longa*; *DA*, *Dendrobium aurantiacum* var. *denneanumis*; *DC*, *Dendrobium chrysotoxum*; *EA*, *Erigeron annuus*; *FL*, *Flavonoids*; *KI*, *KIOM-79*; *OT*, *Ocimum tenuiflorum*; *VK*, *Vitamin K*; *TP*, *Tephrosia purpurea*; *ZM*, *Zea mays*; *MA*, *Matteuorienate A*; *CD*, *Caesalpinia digyna*; *CO*, *Cornus officinalis*; *MC*, *Morinda citrifolia*; *SM*, *Salvia miltiorrhiza*; *FV*, *Foeniculum vulgare*; *PM*, *Pueraria montana*; *DL*, *Danshenol*; *C Ar*, *Citrus aurantium*; *PE*, *Phyllanthus emblica*.

Lipids peroxidation is also a reason of age related cataract. This process has a negative impact on lipid–lipid and lipid–protein interactions. Research has shown high levels of hydroperoxides, oxy derivatives, and diene conjugates of phospholipid fatty acids in the aqueous humor of cataract patients. Also, studies have reported high levels of oxidation products of linoleic acid in patients with early cataract (Bai et al., 2015). A schematic representation of oxidative stress mechanisms involved in cataract etiology and action mechanisms of several medicinal plants with conducted pharmacological studies for the treatment of cataract are presented in Figure 1.

Polyol pathway is associated with diabetic cataract. Enzymes implicated in the polyol pathway, sorbitol dehydrogenase and AR are responsible for the conversion of glucose to fructose. Sorbitol, an intermediate compound, was found to produce cell lesions by modifying the membrane permeability. Therefore, accumulation of sorbitol leads to osmotic stress, collapse, and liquefaction of lens fibers resulting in loss of lens transparency (Pollreisz and Schmidt-Erfurth, 2010; Hashim and Zarina, 2012). AR converts glucose to sorbitol, dependent to NADPH. As a consequence, the level of NADPH decreases, also having a negative impact on the glutathione activity and the antioxidant system. *In vivo* and *in vitro* studies have shown that by inhibiting the activity of AR, the progression to cataract in patients with diabetes is reduced (Kim et al., 2011a; Ramana, 2011). The glycosylation pathway has also been linked to diabetic cataract. Excessive glucose level induces the glycation of proteins, generating superoxide radicals and AGEs in the process. Recent studies suggest that there is interdependence between the oxidative stress and polyol pathway, through AR and iNOS, responsible for the nitric oxide production during oxidation (Snow et al., 2015; Li et al., 2017) (Figure 1).

**FIGURE 2:**
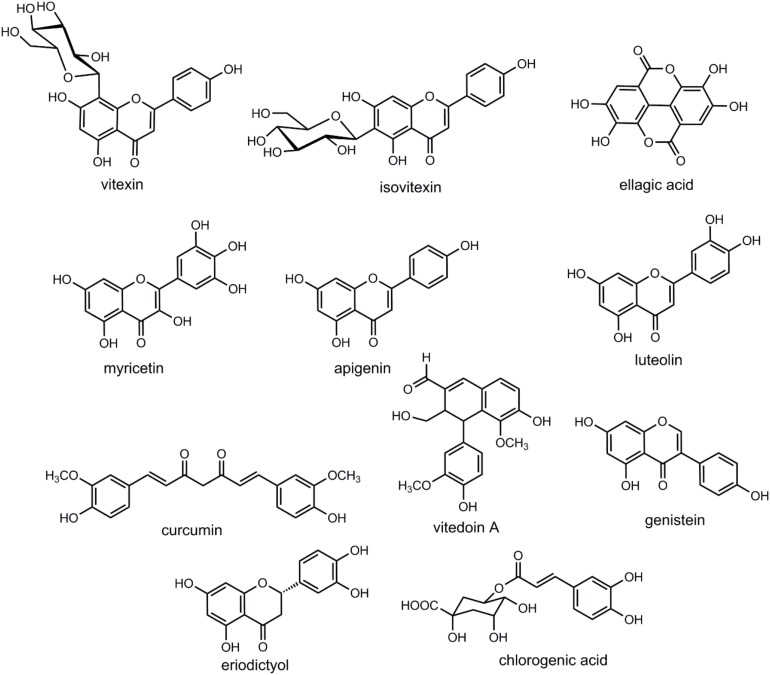
Chemical structures of some of the relevant natural products discussed in the context of cataract treatment.

Here, we present details of plants evaluated against cataract with discussion of their possible mechanism of action (Figure 1 and Tables 1–4). Some important chemical structures of the natural products that are used against cataract or found in plants used in the management of cataract are also presented at Figure 2 (not all chemical structures are presented). We categorized the plants based upon the models evaluated. Table 1 describes the natural products used against cataract evaluated on selenite/sodium selenite induced cataracts, in Table 2 natural products used against cataract on preventive photooxidative damage is described, Table 3 deals with the natural products used against cataract on sugar-induced lens opacity/Streptozotocin induced diabetic cataract/galactose or glucose induced/ZDF models, in Table 4 AGEs-BSA crosslinking inhibition assay and lens AR activity models are described, and Table 5 describes the natural products used against cataract on hydrogen peroxide and naphthalene induced cataract and other miscellaneous models.

**TABLE 1:** Medicinal plants/natural products used against cataract on Selenite/sodium selenite induced cataract models, and suggested/possible mechanisms of action.

**Plant (with part used)/natural product**	**Doses, concentrations and characteristics of extracts**	**Suggested/possible mechanism of action**	**References**
*Alangium salviifolium* (L.f.) Wangerin (Syn. *Alangium lamarckii* Thwaites) (Leaves)	Alcoholic extract at an increasing concentration between 0 and 300 μg/mL (IC${}_{50}$ 106 ± 5.11 μg/mL)	Exhibit significant inhibitory effects on aldose reductase (AR) in the rat lens *in vitro*.	Kumar et al.,2011
*Allium cepa* L. (Tuber/bulb)	50% diluted juice	Prevention of selenite-induced cataract formation by increase in superoxide dismutase (SOD) and total antioxidant level and activities of glutathione peroxidase (GPX) in lens through instillation of juice in rat eyes.	Javadzadeh et al.,2009b
*Allium sativum* L. (Tuber/bulb)	Aqueous extract, 1 mL/kg body weight	Free radical scavenging activity (FRSA), antioxidant properties and associated with increased TA level, SOD and GPX activities in the lens.	Javadzadeh et al.,2009a
*Aralia elata* (Miq.) Seem. (Cortex)	Aqueous extract at 1 and 10 mg/mL (IC${}_{50}$ 11.3 μg/mL)	Inhibit AR and antioxidant activity.	Chung et al.,2005
*Brassica oleracea* L. var. *italica* Plenck (Edible part)	Flavonoid fraction	Maintains antioxidant status, ionic balance via Ca^2+^ ATPase pump, inhibits calpain activation, lipid peroxidation, and protein insolubilization.	Vibin et al.,2010
*Caesalpinia digyna* Rottler (Roots)	Alcoholic extract at an increasing concentration between 0 and 200 μg/mL (IC${}_{50}$ 46.29 ± 11.17 μg/mL)	AR inhibition.	Kumar et al.,2011
Caffeic acid phenethyl ester	Caffeic acid phenethyl ester, subcutaneous	Suppressed cataract formation in rats by antioxidant property.	Doganay et al.,2002
*Camellia sinensis* (L.) Kuntze (Leaves)	Extract (1 L of the solution contains 100 g of green tea, 400 mL of purified water and 535 mL of strong alcohol), intraperitoneally	Antioxidant activity.	Gupta et al.,2002
*Cassia fistula* L. (Fruit pulp)	Sequential water, ethanol and chloroform extracts	AR inhibition.	Gacche and Dhole,2011a
*Cochlospermum religiosum* (L.) Alston (Leaves)	Isolated isorhamnetin-3-glucoside, 50 μg/mL	Retardation of selenite cataract *in vitro* via preventing oxidative stress, calcium accumulation and preclusion of lipid peroxidation.	Gayathri Devi et al.,2010
*Coffea arabica* L.	1 mL of Instant black coffee	Decreased level of total nitric oxide, tumor necrosis factor-α (TNF- α), Ca-ATPase, superoxide dismutase, interleukin (IL)-1β, preserved enzyme antioxidants and lens proteins.	El Okda et al.,2016
*Crataegus pinnatifida* Bunge (Leaves)	Total flavonoids fraction	Inhibition of AR, FRSA.	Wang et al.,2011
*Crocus sativus* L. (Stigmas)	Hydroalcoholic extract, intraperitoneal injections of saffron extract (60 mg/kg body weight)	Reinforcement of antioxidant condition, inhibits lipid peroxidation intensity, and inhibition of aqueous-soluble fraction of lens proteolysis.	Makri et al.,2013
*Curcuma longa* L. (Rhizomes)	Curcumin (200 μM)	Oxidative stress inhibition and attenuation to cataract formation, ameliorated calcium-induced proteolysis.	Manikandan et al., 2009; Liao et al.,2016
*Cyanthillium cinereum* (L.) H. Rob. (Syn. *Vernonia cinerea* Less.) (Leaves)	Isolated lupeol from flavonoid fraction that showed an IC${}_{50}$ 30 μg/mL against DPPH	Protection against formation of nuclear opacity in selenite-treated Sprague Dawley rat pups.	Asha et al.,2016
*Dregea volubilis* (L.f.) Benth. ex Hook.f. (Leaves)	Isolated drevogenin D, 50 μg/mL	Antioxidant activity (affecting glutathione peroxidase, superoxide dismutase, catalase, and glutathione reductase), raises reduced glutathione and protein sulfhydryl levels, and decreases the lipid peroxidation levels.	Biju et al.,2007
Ellagic acid	Ellagic acid 200 mg/kg body weight, *i.p.*	Inhibition of lipid peroxidation and maintains antioxidant defense system.	Sakthivel et al.,2008
*Emilia sonchifolia* (L.) DC. ex DC. (Whole plant)	Flavonoid fraction 1.0 mg/kg body weight, *i.p.*	Antioxidant activity.	Lija et al.,2006
*Enicostemma hyssopifolium* (Willd.) Verd. (Aerial parts)	*C*-glycosidic flavonoid including extract (IC${}_{50}$ 1.62; bitter fraction 2.40, Swertiamarin 7.59 and Swertisin 0.71 μg/mL)	AR inhibition.	Patel and Mishra,2009
*Eucalyptus deglupta* Blume (Not given)	Ethanolic extract	AR inhibition.	Guzman and Guerrero,2005
*Ginkgo biloba* L. (Egb761)	Extract 761 (0.35% 100 mg/kg body weight)	Prevents depletion of antioxidant enzymes, reduces oxidative stress, inhibition of lipid peroxidation and suppression of the TGF-β2/Smad pathway activation.	Lu et al., 2014; Cao et al.,2015
*Jacobaea maritima* (L.) Pelser & Meijden [Syn. *Cineraria maritima* (L.) L.] (Aerial parts)	Ethanolic extract (300 μg/mL)	Increase in the activity of antioxidant enzymes and increase in the level of reduced glutathione in lens, reduces free radical generation.	Anitha et al.,2011, 2013
*Vitex negundo* L. (Leaves)	Flavonoids	Maintenance of antioxidant status, by inhibition of ROS generation/lipid peroxidation in lens.	Rooban et al.,2012
*Moringa oleifera* Lam. (Leaves)	Flavonoid fraction 2.5 μg/g body weight	Improvement of total antioxidant capability in lens, prevention of protein oxidation and lipid peroxidation.	Sasikala et al.,2010
*Origanum vulgare* L. (Upper crust of beans)	Hydroethanolic extract (70%), 2 g/kg	Averts selenite-induced cataract through its antioxidant property.	Dailami et al.,2010
*Phyllanthus emblica* L. (Syn. *Emblica officinalis* Gaertn.) (Fruits)	Aqueous extract, 26.19 mg/kg	Inhibition of sodium selenite induced cataract in rats though antioxidant property.	Nair et al.,2010
*Pleurotus ostreatus* (Jacq. ex Fr.) P.Kumm. (Mushroom)	Ethanolic extract 250 μg/mL	Reduction of lipid peroxidation and increase in antioxidant enzymes.	Isai et al.,2009
Rutin	Rutin	Alteration in protein profile and insolubilization of soluble protein.	Sasikala et al.,2013
*Senna tora* (L.) Roxb. (Syn. *Cassia tora* L.) (Leaves)	Ethyl acetate fraction having anthraquinones and flavonoids, 5 μg/g body weight	Prevention of cytoskeletal protein denaturation in the lens, improvement of antioxidant capacity, and reduction in free radical generation.	Sreelakshmi and Abraham,2016
*Spathodea campanulata* P.Beauv. (Flowers)	Exudate, 0.1 and 0.2 mg/mL	Counteracts cataract by antioxidant activity.	Gbemisola et al.,2014
*Syzygium malaccense* (L.) Merr. & L.M.Perry (Not mentioned)	Ethanolic extract	AR inhibition.	Guzman and Guerrero,2005
*Tagetes erecta* L. (Flowers)	Lutein and its ester at doses of 4, 40, and 400 mg/kg body weight	Antioxidant activity.	Harikumar et al.,2008
*Tephrosia purpurea* (L.) Pers. (Whole plant)	Flavonoid rich fraction (40 mg/kg) or alcohol extract (300 mg/kg)	Maintenance of the antioxidant status and prevention of protein oxidation and lipid peroxidation in lens.	Bhadada et al.,2016a
*Trigonella foenum-graecum* L. (Seeds)	Lyophilized aqueous extract, (25, 50, and 100 μg/mL)	Antioxidant.	Gupta et al.,2010b
Triphala [An Ayurvedic formulation consisting of *Emblica officinalis* Gaertn., *Terminalia chebula* Retz., and *Terminalia bellirica* (Gaertn.) Roxb.]	Aqueous extract at 25, 50, and 75 mg/kg body weight *i.p.*	Restoration of GSH and reduced malondialdehyde levels. Substantial restoration in antioxidant enzymes activities like glutathione peroxidase, superoxide dismutase, catalase, and glutathione-*s*-transferase.	Gupta et al.,2010a
*Vaccinium corymbosum* L. (Leaves)	Decoctions (centrifuged, filtered, lyophilized), and dry extract, dissolved in sterilized normal saline, 100 mg/kg	Direct and indirect inhibition of lens calpains, anti-oxidant and chelating properties.	Ferlemi et al.,2016
*Vitex negundo* L.	Flavonoids	Enhancement of antioxidant enzymes, maintains ionic balance and reduces the lens oxidative stress, prevention of changes in lens protein, loss of chaperone property, changes in lens structure, protective effect against oxidative damage.	Rooban et al.,2009, 2010, 2011
*Vitex negundo* L. (Leaves)	Luteolin	Maintenance of antioxidant status *via* reducing ROS generation/lipid peroxidation in lens.	Rooban et al.,2012
*Vitis vinifera* L. (Seed extract)	Proanthocyanidin/procyanidin-rich extract	Oxidative stress inhibition, suppression of lipid peroxidation, and free radicals and activation of inducible nitric oxide synthase (iNOS), and calpain II in lenses. Improvement of the antioxidant defense mechanisms of the lens.	Yamakoshi et al., 2002; Durukan et al., 2006; Zhang and Hu, 2012; Mani Satyam et al.,2014
*Withania somnifera* (L.) Dunal (Procured extract)	Aqueous extract, 25–300 μg/mL	Inhibits lens AR activity.	Halder et al.,2003

**TABLE 2:** Medicinal plants/natural products used against cataract on preventing photo-oxidative damage.

**Plant (with part used)/ natural product**	**Doses, concentrations and characteristics of extracts**	**Suggested/possible mechanism of action**	**References**
Astaxanthin	Astaxanthin (0–1 mM)	Prevention of cataract through protection of lens from oxidative insults and degradation by calcium-induced calpain.	Wu et al.,2006
*Citrus × aurantium* L. (Peel)	Methanol-water extract, 100 and 200 mg/kg body weight	Delay in onset and maturation of naphthalene induced cataract vis prevention of the photo-oxidative damage produced by naphthalene.	Umamaheswari et al.,2011
*Ginkgo biloba* L. (Leaves)	Standardized EGb761 extract (24% flavonol glycoside and 6% terpene lactones)	Protection from radiation induced cataracts in rat lens *via* antioxidant property.	Ertekin et al.,2004
Lutein and Zeaxanthin	Lutein and Zeaxanthin	Protection of eye from oxidative stress and high-energy photons of blue light.	Moeller et al.,2000

**TABLE 3:** Medicinal plants/natural products used against cataract on sugar-induced lens opacity/streptozotocin induced diabetic cataract/galactose, glucose and xylose induced/Zucker diabetic fatty (ZDF) aldose reductase rat models and possible mechanisms of action.

**Plant (with part used)/natural product**	**Doses, concentrations and characteristics of extracts**	**Suggested/possible mechanism of action**	**References**
*Aegle marmelos* (L.) Corrêa (Leaves)	Chloroform extract 150 mg and 300 mg/kg body weight, p.o.	Increases glutathione, catalase and superoxide dismutase, inhibits lens AR and decreases osmotic stress.	Panaskar et al., 2013; Sankeshi et al.,2013
*Allium sativum* L. (Bulb)	Methanolic extract, 0.25 and 0.5 g/kg body weight, by forcible gut feeding	Antioxidant activity.	Raju et al.,2008
*Angelica dahurica* (Hoffm.) Benth. & Hook.f. ex Franch & Sav. (Roots)	Ether extract (100 μg/mL) (Byakangelicin)	Suppression of galactose induced cataract formation in diabetic rats via AR inhibiting property.	Shin et al.,1994, 1998
*Buddleja officinalis* Maxim. (Flowers)	Apigenin	Inhibiting rat lens AR activity.	Matsuda et al.,1995
*Azadirachta indica* A. Juss. (Not mentioned)	Aqueous extract, 25–300 μg/mL	Inhibits lens AR activity.	Halder et al.,2003
*Biophytum sensitivum* (L.) DC. (Leaves)	Sequential water, ethanol and chloroform extracts	AR inhibition and antioxidant action.	Gacche and Dhole,2011a
*Brassica juncea* (L.) Czern. (Leaves)	Aqueous extract, 250 and 500 mg/kg	Effective activity against hyperglycemia induced oxidative and osmotic stress.	Valavala et al.,2011
*Brickellia arguta* B. L. Rob (Not mentioned)	Ethanolic extract	AR inhibition.	Guzman and Guerrero,2005
*Caesalpinia bonduc* (L.) Roxb. (Seeds)	Sequential water, ethanol and chloroform extracts	AR inhibition and antioxidant.	Gacche and Dhole,2011a
*Cassia fistula* (L.) (Fruit pulp)	Sequential water, ethanol and chloroform extracts	AR inhibition and antioxidant.	Gacche and Dhole,2011a
*Catharanthus roseus* (L.) G.Don (Leaves)	Sequential water, ethanol and chloroform extracts	Inhibiting AR activity and antioxidant action.	Gacche and Dhole,2011b
Chlorogenic acid	Chlorogenic acid (0.7–2.8 mM)	Inhibiting AR activity in galactose fed rats.	Kim et al.,2011a
*Chromolaena odorata* (L.) R.M.King & H.Rob. (Leaves)	Ethanol extract (200 and 400 mg/kg)	Decrease of oxidative stress.	Onkaramurthy et al.,2013
*Corydalis turtschaninovii* Besser (Tuber)	Methanolic extract of the alkaloidal component (10–200 μg/mL) containing dehydrocorydaline	Inhibiting AR activity.	Kubo et al.,1994
*Curcuma longa* L. (Rhizome)	Aqueous extract, 25–300 μg/mL	Prevents *in vitro* cataract *via* AR inhibitory activity.	Halder et al.,2003
*Dendrobium chrysotoxum* Lindl. (Stems)	Gigantol	Inhibition of AR and AR gene expression.	Wu et al.,2017
*Dendrobium aurantiacum* (F. Muell.) F. Muell. var. *denneanumis* (Kerr) Z.H.Tsi (Stems)	Gigantol	Attenuation in increase of AR, inducible nitric oxide synthase (iNOS) expression and opacification of rat lenses.	Fang et al.,2015
*Eclipta prostrata* (L.) L. [Syn. *Eclipta alba* (L.) Hassk.] (Whole plant)	Ethanolic extract (flavonoids)	Inhibition of AR	Jaiswal et al.,2012
*Erigeron annuus* (L.) Pers. (Flowers)	Isolated phenolic compounds	Inhibition of cataract *via* inhibiting protein glycation and AR in rat lens.	Jang et al.,2008
*Eugenia cordata* (Sw.) DC.var. *sintenisii* (Kiaersk.) Krug & Urb. (Not mentioned)	Ethanolic extract	Inhibition of AR.	Guzman and Guerrero,2005
*Ficus glomerata* L. (Fruits)	Sequential water, ethanol, and chloroform extracts	AR inhibition and maintaining of lens opacity.	Gacche and Dhole,2011b
*Marsdenia sylvestris* (Retz.) P.I.Forst. [Syn. *Gymnema sylvestre* (Retz.) R. Br.] (Leaves)	The polyol Conduritol A	Inhibition of AR.	Miyatake et al.,1994
*Thymus vulgaris* L. (Leaves)	Methanolic extract and isolated compounds (Eriodictyol)	Suppression of the advanced glycation end products levels and fructosamines of albumin.	Morimitsu et al.,1995
Genistein	Genistein	Increase connexin (Cx) 43 expression.	Huang et al.,2007
*Zingiber officinale* Roscoe (Rhizomes)	Powder	Suppressing lens galactitol accumulation.	Saraswat et al.,2010
*Hydrocotyle bonariensis* Comm. ex Lam. (Leaves)	Aqueous extract, 500 and 1,000 mg/kg	Reduction in lens protein insolubilization, lens peroxidation and increase in the antioxidant status of the lens.	Ajani et al.,2009
*Justicia adhatoda* L. (Syn. *Adhatoda vasica* Nees.) (Procured extract)	Sequential water, ethanol and chloroform extracts	AR inhibition and antioxidant action.	Gacche and Dhole,2011a
KIOM-79	(80% ethanol extract of parched Puerariae Radix, gingered). (Magnoliae cortex, Glycyrrhizae Radix and Euphorbiae Radix) (*Magnolia officinalis*, *Pueraria lobata*, *Glycyrrhiza uralensis*, *Euphorbia pekinensis*) (0–1,000 μg/mL)	AR inhibition. KIOM-79, an Inhibitor of AGEs–Protein Cross-linking, Prevents Progression of Nephropathy in Zucker Diabetic Fatty Rats.	Kim et al.,2011b
*Magnolia fargesii* (Finet & Gagnep.) W. C. Cheng (Flower buds)	Isolated scopoletin and tiliroside	Inhibition of rat lens aldose reductase (RLAR) activity; *ex vivo* cataractogenesis of rat lenses induced by xylose was inhibited by scopoletin.	Lee et al.,2010
*Mangifera indica* L.	Ethanolic extract	AR inhibition and antioxidant activity.	Guzmán and Guerrero,2005
*Miyamayomena koraiensis* (Nakai) Kitam. (Syn. *Aster koraiensis* Nakai) (Korean starwort) (Aerial part)	Extract of 100 and 200 mg/kg	Delay in the progression of lens opacification during the early diabetic cataractogenesis.	Kim et al.,2009
*Momordica charantia* L. (Fruits)	Aqueous and ethanolic extracts, 200 and 400 mg/kg	Prevention of experimental diabetic cataract through reduction of plasma glucose levels.	Rathi et al.,2002
*Ocimum tenuiflorum* L. (Syn. *Ocimum sanctum* L.) (Leaves)	Aqueous extract, 25–300 μg/mL	Prevents *in vitro* cataract by virtue of its aldose reductase inhibitory activity.	Halder et al.,2003
Peonidin-3-glucoside	Peonidin-3-glucoside	Inhibits lens AR.	Morimitsu et al.,2002
*Phyllanthus emblica* L. (Syn. *Emblica officinalis* Gaertn.) (Fruits)	Isolated β-glucogallin (0–40 μM)	Inhibition of AKR1B1.	Puppala et al.,2012
*Pterocarpus marsupium* Roxb. (Bark)	Aqueous extract, 2 g/kg	Decreased opacity index.	Vats et al.,2004
*Pueraria montana* (Lour.) Merr. var. *lobata* (Willd.) Sanjappa & Pradeep. (Roots)	Puerariafuran isolated from methanoilc extract	Inhibition of rat lens AR.	Kim et al.,2010
Rutin	Rutin (10–100 μM)	Inhibits advanced glycation end products formation by prevention of dicarbonyls formation.	Muthenna et al.,2012
Silybin	Silybin, 231 mg/day for 4 weeks	Reductions in the erythrocytic sorbitol level which lead to formation of glycation end products.	Zhang et al.,1995
*Silybum marianum* (L.) Gaertn. (Seeds)	Silymarin 200 mg/kg/d, from extract	Antioxidative activity and increase in lens GSH and decrease in lipid peroxides (LPO) levels.	Fallah Huseini et al.,2009
*Syzygium cumini* (L.) Skeels (Syn. *Eugenia jambolana* Lam.) (Kernels)	Aqueous and ethanolic extracts, 200 and 400 mg/kg	Significant reduction of plasma glucose.	Rathi et al.,2002
*Syzygium nervosum* A.Cunn. ex DC. [*Cleistocalyx operculatus* (Roxb.) Merr. & L.M.Perry] (Dried flower buds)	Aqueous extract, 500 mg/kg bw/day	Indirect antihyperglycemic effect, decreases the levels of glucose, sorbitol, and fructose in diabetic rat lenses.	Mai et al.,2010
*Tephrosia purpurea* (L.) Pers. (Whole plant)	Flavonoid rich fraction, 40 mg/kg/day, p.o, whole plant	AR enzyme inhibition and anti-oxidant activity.	Bhadada et al.,2016b
*Theobroma cacao* L. (Cacao liquor)	Crude polyphenol fraction (0.5% with diet) (Cyanidin)	Inhibits lens AR.	Osakabe et al.,2004
*Tinospora sinensis* (Lour.) Merr. [Syn. *Tinospora cordifolia* (Willd.) Miers] (Procured stem extract)	Aqueous and ethanolic extracts, 200 and 400 mg/kg	Prevention of retinal oxidative stress, restoration of antioxidant enzyme levels and reduction in the angiogenic markers, vascular endothelial growth factor (VEGF) and protein kinase C (PKC) that are increased in diabetic retina.	Rathi et al., 2002; Rajalakshmi et al., 2009; Agrawal et al.,2012
Triphala Ghrita	It’s an Ayurvedic formulation containing gallic acid	Delay in the onset and progression of galactose induced cataract through antioxidant activity.	Mahajan et al.,2011
Vitamin K	Vitamin K	Lens Ca^2+^ homeostasis modulation and inhibition of osmotic and oxidative stress.	Sai Varsha et al.,2014
*Zea mays* L. (Seed)	Hydroalcoholic extract, 2, 10, and 50 mg/mL	Decline in oxidative stress and inhibition of aldose reductase.	Thiraphatthanavong et al.,2014
*Zingiber officinale* Roscoe (Rhizomes)	Powder	Reduction in the carbonyl stress, inhibition of osmotic stress by reduction in the activity of the polyol pathway, oxidative stress prevention.	Saraswat et al.,2010

**TABLE 4:** Medicinal plants/natural products used against cataract on advanced glycation end products (AGE)- BSA cross-linking inhibition assay and lens aldose reductase activity models and possible mechanisms of action.

**Plant (with part used)/natural product**	**Doses, concentrations and characteristics of extracts**	**Suggested/possible mechanism of action**	**References**
*Caesalpinia digyna* Rottler (Roots)	Alcoholic extract at an increasing concentration between 0 and 200 μg/mL (IC${}_{50}$ 46.29 ± 11.17 μg/mL)	AR inhibition and antioxidant action.	Kumar et al.,2011
*Cinnamomum verum* J.Presl (Bark)	Ethanolic extract fractions containing Procyanidin-B2, 1–3 mg	AGE inhibition of eye lens proteins under *in vitro* conditions and inhibition of the formation of glycosylated hemoglobin in human blood in *ex vivo* conditions.	Muthenna et al.,2013
*Cornus officinalis* Siebold & Zucc. (Seeds)	EtOAc-soluble fraction (Galloyl glucoses)	Inhibition of formation of AGE, AGE- BSA cross-linking, and RLAR.	Lee et al.,2011
*Erigeron annuus* (L.) Pers. (Leaves and stems)	3,5-Di-*O*-caffeoyl-epi-quinic acid isolated from methanolic fraction, 5 μM	Inhibition of AGEs, AGEs-BSA cross-linking to collagen, RLAR formation, and prevention of lenses opacification.	Jang et al.,2010b
Flavonoids	Chrysin, apigenin, and baicalein	Inhibition of glycation, glycation induced lens opacity, AGEs, AR and lens protein aggregation.	Patil et al.,2016
*Hybanthus enneaspermus* (L.) F.Muell. (Whole plant)	Different fractions from ethanolic extract, 0–300 μg/mL	Not clearly described.	Patel et al.,2012
*Magnolia biondii* Pamp. [Syn. *Magnolia fargesii* (Finet & Gagnep.) W.C.Cheng] (Flower buds)	Isolated scopoletin and tiliroside	RLAR inhibition.	Lee et al.,2010
*Onoclea orientalis* (Hook.) Hook. (Syn. *Matteuccia orientalis* Trev.) (Rhizomes)	Isolated compound Matteuorienate A, Matteuorienate B from the methanolic extract	AR inhibition.	Kadota et al.,1994
*Morinda citrifolia* L. (Fruits)	Sequential water, ethanol, and chloroform extracts	AR inhibition and free radical scavenging activity.	Gacche and Dhole,2011b
*Onoclea orientalis* (Hook.) Hook. (Syn. *Matteuccia orientalis* Trev.) (Rhizomes)	Isolated compound from the methanolic extract Matteuorienate C	AR inhibition.	Basnet et al.,1995
*Platycodon grandiflorus* (Jacq.) A.DC. (Flowers)	Isolated compounds from ethyl acetate soluble fractions [apigenin, luteolin, luteolin-7-*O*-β-D-glucopyranoside, luteolin-7-*O*-(6″-*O*-acetyl)-β-D-glucopyranoside, apigenin-7-*O*-β-D-glucopyranoside, apigenin-7-*O*-(6″-*O*-acetyl)-β-D-glucopyranoside, isorhamnetin-3-Oneohesperidoside, 4-*O*-caffeoylquinic acid, chlorogenic acid methyl ester, 4-*O*-β-D-glucopyranosyl caffeic acid]	Substantial inhibition of AGEs formation and RLAR.	Jang et al.,2010a
*Salvia miltiorrhiza* Bunge (Roots)	Constituents of methanolic extract Danshenol A Danshenol B, (-)-Danshexinkun A, Dihydrotanshinone I, Tanshinone IIA	AR inhibition.	Tezuka et al.,1997

**TABLE 5:** Medicinal plants/natural products used against cataract on hydrogen peroxide- and naphthalene induced cataract and other miscellaneous models and possible mechanisms of action.

**Plant (with part used)/ natural product**	**Doses, concentrations and characteristics of extracts**	**Suggested/possible mechanism of action**	**References**
*Abies pindrow* (Royle ex D.Don) Royle (Leaves)	Aqueous extract (5–20 mg/mL)	Inhibition of free radical generation.	Dubey et al.,2015b
*Acorus calamus* L. (Roots)	Methanolic extract and β-asarone	Significantly retarded experimental hydrogen peroxide induced cataractogenesis.	Kumar and Singh,2011
*Cistanche deserticola* Y.C.Ma and SkQ1 (Stolons)	Fraction	Increase of the lens protein solubility and destroying of large protein aggregates, antioxidant action leads to elevation of tryptophan and kynurenine levels in the lens.	Snytnikova et al.,2012
*Elaeagnus rhamnoides* (L.) A.Nelson (Syn. *Hippophae rhamnoides* L.) (Leaves)	Aqueous extract, 100–1,000 μg/mL	Regulation of oxidative stress and promotion of antioxidant systems.	Dubey et al.,2016
*Erythrina stricta* Roxb. (Leaves)	Hydromethanolic extract (fractions), 200 mg/kg	Antioxidant activity, prevented the peroxidative damage caused by naphthalene.	Umamaheswari et al.,2010
*Foeniculum vulgare* Mill. (Fruits)	Petroleum ether fraction, 10 mg/kg, twice daily	AR reduction and antioxidant action.	Dongare et al.,2012
L-arginine	L-arginine	Blocking of carbonyl stress in the lens.	Fan et al.,2011
*Luffa cylindrica* (L.) M.Roem. (Fruits)	Standardized extract, 5–30 μg/mL	Protection of lens proteins from oxidative damage.	Dubey et al.,2015a
*Nigella sativa* L. (Seeds)	Oil	Inhibiting of RNS generation, antioxidant action, and FRSA.	Taysi et al., 2015; Demir et al.,2016
*Ocimum tenuiflorum* L. (Leaves)	Aqueous extract, 150 μg/mL	FRSA.	Halder et al.,2009
*Pueraria montana* var. *lobata* (Will) Sanjappa & Pradeep [Syn. *Pueraria lobata* (Willd.) Ohwi] (Roots)	Puerariafuran isolated from methanolic extract	Inhibition of AR, xylose-induced lens opacity, and the oxidation in lenses.	Kim et al.,2010
*Vitis vinifera* L. (Seed)	Extract constituting of 95% proanthocyanidins	Attenuates cell signaling, cell migration and inflammation, prevention of oxidative stress, inhibition of H_2_O_2_-induced phosphorylation of the p38 and c-Jun N-terminal kinase.	Jia et al.,2011

Like in case of any other disease conditions, medicinal plants are being used in management of various eye ailments from ancient times. Medicinal plants are used in case of cataract, eye infections, conjunctivitis, eye dryness, and other eye disorders in many countries including India (Sandhu et al., 2011; Das et al., 2013; Rothe and Maheshwari, 2016), Bangladesh (Yusuf et al., 2006; Das et al., 2007, 2013), Nepal (Manandhar, 2002; Acharya and Pokhrel, 2006; Gewali, 2011; Watanabe et al., 2013), Sudan (Khalid et al., 2012), Tanzania (Maregesi et al., 2016), South Africa (Pendota et al., 2008), and many other regions of the world.

**FIGURE 3:**
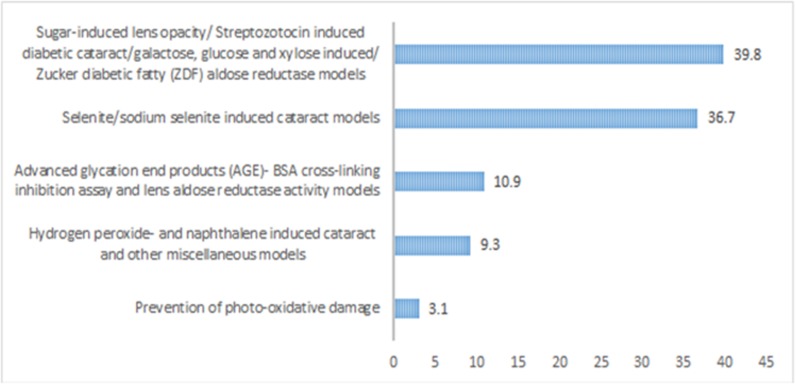
Percentage of different models used for evaluation of anticataract activity of plants/natural products.

The literature analysis revealed that the sugar-induced or diabetic cataract models were the highest used models which were applied for the evaluation of around 39.84% of the plants/natural products. It was followed by selenite/sodium selenite induced cataract which is another common model of evaluation of cataract, and it accounts for around 36.71% of the plants/natural products. AGE-BSA crosslinking inhibition assay was used for the evaluation of 10.93%, and hydrogen peroxide and naphthalene induced cataract was account for evaluation of around 9.38% of the plants (Figure 3). In most of the cases especially for the diabetic cataract models, it was found that different antioxidant parameters like soluble protein, reduced glutathione, superoxide dismutase, lipid peroxidation were used (Bhadada et al., 2016b). Inhibition of AR was found as the most common hypothesis in these models (Bhadada et al., 2016b). Uses of *in silico* studies were also found common in some studies to explore the binding mode of the phytochemicals with the aldose reductase enzyme (Bhadada et al., 2016b; Patil et al., 2016).

In most of the studies, rats or rat pups lens were utilized as the model (Bhadada et al., 2016b; El Okda et al., 2016; Ferlemi et al., 2016; Sreelakshmi and Abraham, 2016) and in some cases fresh goat eyeballs were also used (Patil et al., 2016). *In vitro* studies were also utilized in large number of experiments. In some studies lens crystalline turbidity assay was used by estimation of lens protein turbidity using homogenized decapsulated porcine lenses which were procured from the local slaughterhouses in some cases (Ferlemi et al., 2016; Liao et al., 2016). Some other important factors in cataractogenesis like UV radiation was also used by researchers, and it was also proposed that some compounds can protect γ-crystallin from UV radiation damage and can act as potential anticataract agents (Liao et al., 2016).

Many of the mentioned plants showed potent anticataract activity in *in vitro* and *in vivo* models. *Vitex negundo* and *Vitis vinifera* were the plants in which sufficient preclinical studies were conducted and they may be of potential clinical use. It is also interesting that *Vitex negundo* was also used in the folk medicine in India (Kulkarni et al., 2008). The genus *Ocimum* was also one such genus which is utilized in folk medicine and was scientifically validated for its anticataract potential. Some other interesting findings were the use of *Pleurotus ostreatus* extract that prevented cataract in 75% of the tested rats (Isai et al., 2009). In a clinical study, although not directly against cataract, silybin improved the peripheral nerve conduction velocity and was reported as an effective aldose reductase inhibitor that can improve the disorder of polyol pathway in non-insulin dependent diabetes patients and prevent chronic complications of diabetes (Zhang et al., 1995) like cataract.

The detailed list of medicinal plants used in the management of cataract as reported in many ethnopharmacological surveys is given in Table 6. Singh et al. (2012) had also listed the medicinal plants used in management of cataract, however, the mechanistic insight was not performed and plants used in the management of cataract available till 2011 were covered (Singh et al., 2012).

**TABLE 6:** Medicinal plants reported globally by different ethnopharmacology/ethnobotanical surveys to be used in the treatment of cataract.

**Plant**	**Formulations and mode of administration**	**Major chemical constituents**	**Country**	**References**
*Abrus precatorius* L. (Fabaceae)	Fresh leaves are squeezed and juice is used as eye drops	Abrine; trigonelline; abruslactone A; hemiphloin and abrusin	India/Tanzania	Ragasa et al., 2013; Garaniya and Bapodra, 2014; Maregesi et al.,2016
*Aloe vera* (L.) Burm. f. (Asphodelaceae)	One drop of leaf juice twice a day is used as eye drop	Anthraquinones; aloe emodin and chrysophanol	Tanzania	Lee et al., 2013; Maregesi et al.,2017
*Barleria prionitis* L. (Acanthaceae)	Leaf juice is used	Phenylethanoid glycoside; barlerinoside along with six known iridoid glycosides	Sri Lanka	Jaiswal et al., 2014; Rajamanoharan,2014
*Bidens pilosa* L. (Asteraceae)	Juice of fresh leaves is used as eye drops.	Phenylheptatriyne; linoleic acid; linolenic acid; friedelin and friedelan-3 beta-ol	Tanzania	Geissberger and Sequin, 1991; Maregesi et al.,2016
*Boquila trifoliolata* (DC.) Decne. (Lardizabalaceae)	Fresh leaves are squeezed and the juice is utilized as eye drops.	Oleanolic acid	Chile	Gusinde, 1936a, 1936b; Silva and Stück,1962
*Breynia vitis-idaea* (Burm.f.) C.E.C.Fisch. (Syn. *Breynia rhamnoides* M. Arg.) (Euphorbiaceae)	Stem exudate is put in the eyes for 2–3 days in the morning	Breynin and breyniaionoside E	India	Parinitha et al., 2004; Meng et al.,2010
*Byttneria herbacea* Roxb. (Malvaceae)	Root paste is used	Not described	India	Biswas et al.,2016
*Chusquea quila* Kunth (Poaceae)	New stems are heated and the juice of the stem is received in a vessel and mixed with breast milk and *Oxalis rosea*	Holocellulose; lignin and α-Cellulose	Chile	Gusinde, 1936a, 1936b; Oliveira et al.,2016
*Citrus limon* (L.) Osbeck (Rutaceae)	Salted lemon juice is used as eye drops.	Linalool; α-humulene; α-pinene and limonene	Tanzania	Golmakani and Moayyedi, 2015; Maregesi et al.,2017
*Coccinia grandis* (L.) Voigt (Cucurbitaceae)	Juice of the stems is dripped into the eyes to treat cataract.	Cephalandrine A and cephalandrine B	Nepal	Gewali, 2011; Manandhar,2002
*Colocasia* sp. (Araceae)	Leaves are cooked and eaten for 2–4 weeks or till curing.	Flavonoids; β-sitosterol and steroids	Bangladesh	Prajapati et al., 2011; Linky et al.,2015
*Commiphora edulis* (Klotzsch) Engl. (Burseraceae)	Fresh latex which was produced on detachment of leaf is applied daily until recovery.	Not described	Tanzania	Maregesi et al.,2016
*Croton caudatus* Geisel. (Euphorbiaceae)	Gum/sap is mixed with mustard oil and applied to eyes.	Bis(2,3-dihydroxypropyl) nonanedioate; 12-*O*- (α-methyl)butyrylphorbol-13-decanoate; 12-*O*- tiglylphorbol-13-decanoate; (9*S*,10*R*,11*E*,13*R*)- 9,10,13-trihydroxyoctadec-11-enoic acid; methyl (9*S*,10*R*,11*E*,13*R*)-9,10,13- trihydroxyoctadec-11-enoate; 4(1H)- quinolinone and 5-hydroxy-2-pyridinemethanol	Bangladesh	Linky et al., 2015; Su et al.,2016
*Croton bonplandianus* Baill. (Euphorbiaceae)	Young stem juice is used as eye drops	Phorbol esters	India	Phillipson, 1995; Islam et al., 2010; Vashistha and Kaur,2013
*Diplolepis geminiflora* (Decne.) Liede & Rapini (Apocynaceae)	The latex secreted when cutting a branch is applied over the eyes	Not described	Chile	Gusinde,1936a, b
*Dolichos trilobus* L. (Leguminosae)	Leaf juice is boiled, cooled and applied.	Doliroside A; phenols and tannins	Tanzania	Hedberg et al., 1983; Bisby,1994
*Duchesnea indica* (Jacks.) Focke (Rosaceae)	Leaf juice is applied	Ellagitannins; ellagic acid glycosides; hydroxybenzoic acid; ellagic acid; hydroxycinnamic acid derivatives, and flavonols	India	Kapkoti et al., 2011; Zhu et al.,2015
*Eryngium paniculatum* Cav. & Dombey ex F. Delaroche (Apiaceae)	Decoction of the root is put in the eyes	(*E*)-anethole; α-pinene; (-)-2,4,4-Trimethyl-3- formyl-2,5-cyclohexadienyl angelate	Chile	Gusinde, 1936a, 1936b; Cobos et al.,2002
*Erythrina indica* Lam. (Papilionaceae)	Juice is put drop-wise in the affected eye	Lectin	India	Konozy et al., 2002; Parinitha et al., 2004; Biswas et al.,2016
*Euphorbia hirta* L. (Euphorbiaceae)	Fresh latex obtained from detached leaves, three drops used for three times a day	Afzelin; quercitrin; myricitrin; rutin; quercetin; euphorbin-A; euphorbin-B; euphorbin-C and euphorbin-D	Tanzania	Kumar et al., 2010; Maregesi et al.,2016, 2017
*Fascicularia bicolor* (Ruiz & Pav.) Mez (Bromeliaceae)	The juice of the young plant parts	Not described	Chile	Gusinde,1936a, b
*Ficus benghalensis* L. (Moraceae)	Milky juice is used	Alkaloids; glycosides, terpenoids; flavonoids; and tannins	Nepal	Acharya and Pokhrel, 2006; Ogunlowo et al.,2013
*Geranium core-core* Steud. (Geraniaceae)	Powdered roots are placed on the eyes	Hexadecanoic acid; hexahydrofarnesyl acetone and tetracosane	Chile	Gusinde, 1936a, 1936b; Radulovic et al.,2011
*Ludwigia hyssopifolia* (G.Don) Exell (Onagraceae)	Juice of this plant along with *Ocimum americanum* L.- Camphor type (2 drops thrice daily for 7–8 days) is given in the eye as a drop.	Piperine	Bangladesh	Yusuf et al., 2006; Das et al.,2007
*Marchantia polymorpha* L. (Marchantiaceae)	Ointment of the crushed plant is prepared, applying it to the eyes	Polymorphatin A; Isorricardin D; 11,1′,13′- trihydroxyisorricardin; 2-[3-(hydroxymethyl)phenoxy]-3-[2-(4-hydroxyphenyl)ethyl]phenol; marchantin J and perrottetin E; 22-hydroxyhopane; 17(21)-hopene; 6α,22-dihydroxyhopane; 20α,22-dihydroxyhopane; 21,22-dihydroxyhopane; 6α, 11α, 22-trihydroxyhopane; 22,28-didroxyhopane; β-sitosterol and daucosterol	Chile	Gusinde, 1936a, 1936b; Fang et al.,2007, 2008
*Microglossa pyrifolia* (Lam.) Kuntze (Asteraceae)	Root juice is used as eye drops	*α*-Humulene and *α*-pinene, Δ^3^-carene, (*E*)-*β*-ocimene and germacrene D	Tanzania	Hedberg et al., 1983; Boti et al.,2007
*Nepenthes khasiana* Hook.f. (Nepenthaceae)	Not described	Droserone; 5-*O-*methyldroserone and naphthoquinones	India	Eilenberg et al., 2010; Dhal et al.,2011
*Nephrolepis biserrata* (Sw.) Schott (Nephrolepidaceae)	Rhizome is scrubbed in the eyes	1β,11α-Diacetoxy-11,12-epoxydrim-7-ene; 1β,6α,11α-triacetoxy-11,12-epoxydrim-7-ene; 1β,3β,11α-triacetoxy-11,12-epoxydrim-7-ene; 9(11)-fernene	Chile	Gusinde, 1936a, 1936b; Bottari et al., 1972; Siems et al.,1996
*Ocimum americanum* L. (Lamiaceae)	Juice of *O*. *americanum* with *Ludwigia hyssopifolia* (two drops thrice daily for 7–8 days) is given in the eye as a drop	1,8-Cineol; camphor; α-pinene and *trans*-α-bergamotene	Bangladesh	Yusuf et al., 2006; Bayala et al.,2014
*Oenothera acaulis* Cav. (Onagraceae)	Stem juice is given in the eye as a drop.	Not described	Chile	Gusinde,1936a, b
*Oxalis corniculata* L. (Oxalidaceae)	Leaf juice is used.	Flavonoids; iso-vitexin; vitexin-2”-*O*-β–D-glucopyranoside; oleic acid; palmitic acid; linoleic acid; linolenic acid and stearic acid	India	Badwaik et al., 2011; Vashistha and Kaur,2013
*Oxalis rosea* Jacq. (Oxalidaceae)	Plant material scrubbed in the eye.	Ascorbic acid; oxalic acid; dehydroascorbic acid; pyruvic acid and glyoxalic acid	Chile	Gusinde, 1936a, 1936b; Montes and Wilkomirsky, 1985; Das,1990
*Phyllanthus amarus* Schum. &Thonn. (Phyllanthaceae)	Fresh leaves are squeezed and juice is utilized as eyes drops, 2–3 drops thrice daily for 7 days.	Amariin	Tanzania	Foo, 1993; Maregesi et al.,2016
*Ribes punctatum* Ruiz & Pav. (Grossulariaceae)	Not described	Cyanidin-3-glucoside; cyanidin-3-rutinoside; delphinidin-3-rutinoside; delphinidin-3-glucoside; 3-caffeoylquinic acid; (epi)-gallocatechin and (epi)-catechin tetramers	Chile	Gusinde, 1936a, 1936b; Jiménez-Aspee et al.,2016
*Rumex usambarensis* (Dammer) (Polygonaceae)	Aerial parts are squeezed and the juice is used as eye drops 2 times daily till recovery.	Chrysophanol, physcion, and emodin	Tanzania	Midiwo and Rukunga, 1985; Maregesi et al.,2016
*Stellaria media* (L.) Vill. (Caryophyllaceae)	Aerial parts are scrubbed in the eyes	2,4,5,7-tetramethyloctane; 6-methylheptyl-3′-hydroxy-2′-methylpropanoate; 2, 2,4-trimethyloctan-3-one; apigenin 6-*C*-β-D- galactopyranosyl-8-*C*-α-L-arabinopyranoside; apigenin 6-*C*-α-L- arabinopyranosyl-8-*C*-α-D-galactopyranoside	Chile	Gusinde, 1936a, 1936b; Hodisan and Sancraian, 1989; Kitanov, 1992; Pande et al., 1995; Hu et al., 2006; Vanhaecke et al., 2008; Sharma and Arora, 2012; Arora and Sharma,2014
*Solanum lycopersicum* L. (Solanaceae)	Fresh leaves are squeezed and the juice is used as eye drops.	Adenosine	Tanzania	Fuentes et al., 2012; Maregesi et al.,2016
*Solanum virginianum* L. (Solanaceae)	Seed is used	Arabinogalactan, glycosides	India	Pattanayak et al., 2012; Raja et al.,2014
*Swietenia macrophylla* King (Meliaceae)	One drop of fresh latex produced from bark is used once daily	Swietemacrophyllanin; catechin and epicatechin	Tanzania	Falah et al., 2008; Maregesi et al.,2016
*Thunbergia grandiflora* (Roxb. ex Rottl.) Roxb. (Acanthaceae)	Bubbles of 1–2 drops of the watery latex from the stem are blown gently into the affected eyes, 3 times a day for 4–5 days	Isounedoside and grandifloric acid	India	Ismail et al., 1996; Dipankar,2012
*Typha angustifolia* L. (Typhaceae)	New stems are applied on the eye	Pentacosanoic acid; β-sitosterol; nonadecanol; naringenin; daucosterol; uracil typhaneoside; nicotinic acid; vanillic acid; succinic acid; thymine; stearic acid propanetriol ester	Chile	Gusinde, 1936a, 1936b; Jia et al., 1986; Liao et al., 1990; Chen et al., 2008; Varghese et al., 2009; Shukla et al.,2013
*Tridax procumbens* (L.) L. (Asteraceae)	Leaf juice is dripped into the eyes to treat cataract	Procumbentin	Nepal	Manandhar,2002
*Vernonia amygdalina* Delile (Asteraceae)	Fresh leaves are squeezed and the juice is used as eyes drops, 2–3 drops are used thrice daily for 7 days	Steroidal saponins; tannins; alkaloids; and flavonoids	Tanzania	Omoregie and Pal, 2016; Maregesi et al.,2017
*Vitex negundo* L. (Lamiaceae)	NA	Vitedoin A; vitedoamine A; vitexdoin A; flavonoids; lignans; and terpenoids	India	Tewar et al., 2013; Shu et al.,2016

**FIGURE 4:**
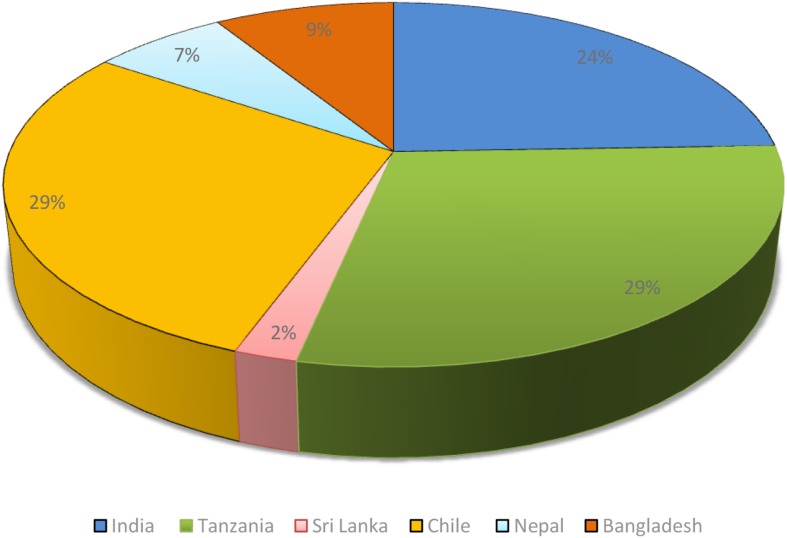
Distribution of plants used in folklore medicine.

It was found that most of the surveys were conducted in different developing countries like Bangladesh, Chile, India, Nepal, and Tanzania (see Figure 4). Apart from the ethnobotanical surveys, several plants used in traditional medicine systems like Ayurveda were also found beneficial for cataract. One such good example of use of Ayurvedic formulation against cataract is the use of *Triphala* which showed good effect against cataract in *in vitro* and *in vivo* (Gupta et al., 2010a; Mahajan et al., 2011) studies and also was evaluated clinically and showed promising results (Bhati and Manjusha, 2015), however, more clinical studies are required involving larger patients for better scientific evidences. Plants used in Ayurveda like *Momordica charantia*, *Eugenia jambolana*, *Pterocarpus marsupium*, and *Trigonella foenum-graecum* prevented cataract development when observed in alloxan diabetic cataract model (Rathi et al., 2002; Vats et al., 2004).

Although, many plants have been utilized in various folklore medical practices, most of them are not scientifically validated. Moreover, some of these traditional practices may be harmful for the eyes as well. For instance, use of latex/sap of some Euphorbiaceae plants like *Euphorbia hirta* and *Croton caudatus* can be dangerous for eyes rather being beneficial. Moreover, sufficient care is obligatory while using the herbal medication for any of the eye diseases, as there is a case study that showed that cataract or development of cataract was aggravated after treatment with some unrevealed herbal medication in a 11 years old patient with atopic dermatitis (Kang et al., 2008).

This survey reveals that selenite/sodium selenite induced cataracts was the preferred model in studies with natural products used against cataract, followed by sugar-induced lens opacity/diabetes induced cataract models, AGE- BSA cross-linking inhibition assay, lens aldose reductase activity models, and hydrogen peroxide-induced cataract, and other miscellaneous models.

Diverse and sometimes complex phytochemicals present in numerous plants possess a broad spectrum of mechanisms for cataract treatment. One of the main mechanisms for the anticataract effect is the antioxidant effect (inhibitory effect on ROS formation). Several plants increase the activity of antioxidant enzymes as well. Some other important mechanisms involved are calpain inhibition, inhibition of lipid peroxidation, amelioration of calcium induced proteolysis, alteration in the protein profiles and the insolubilization of soluble proteins, attenuation in the inducible nitric oxide synthase expression, and AR inhibition.

For development of ocular drug delivery, various factors should be considered which affect the bioavailability of the ocular drugs. These factors include pH, structural forms of the drug, osmolarity, viscosity, tonicity, and the salt form of the drug (Goodman, 1996). Increasing scientific evidence clearly reveals that natural products have the potential to combat cataract at different levels but at the same time critical evaluation for the safety and toxicity profiles are required. Despite of potential evidence for the significance of natural products against cataract, the possible clinical trials are still lacking.

## Conclusion

Ethnopharmacological evaluation of the medicinal plants used for cataract treatment could be a beneficial approach for the development of potential natural product-based therapies against cataract. In this work we have analyzed over 120 papers and found that around 44 medicinal plants/natural products are used for cataract management in different traditional and folk medicine systems. Possible mechanisms of around 118 plants/natural products (including repetition in different models) are also studied. It is interesting that most of the ethnobotanical survey studies are reported from many developing countries like Bangladesh, Chile, India, Nepal, and Tanzania. The medicinal plants are not only utilized in the folk medicine but many of the plants are also mentioned for cataract management in the traditional systems of medicine like in Ayurveda, traditional Chinese medicine and in Korean tradition medicine. However, it is still of utmost importance to document more comprehensively the plants utilized in the traditional medicine and rigorous preclinical and clinical studies are required to validate the use of such plants.

It was also notable that some combinations of plants were also used such as KIOM-79 which is a mixture of ethanol extract (80%) of parched Puerariae Radix, gingered Magnoliae Cortex, Glycyrrhizae Radix, and Euphorbiae Radix is used in Korean tradition medicine. Another such important combination formulation is ‘Triphala’ which is widely used Ayurvedic formulation in India containing fruits of *Emblica officinalis* Gaertn., *Terminalia chebula* Retz., and *Terminalia belerica* (Gaertn.) Roxb. However, many of the plants used in traditional medicines are not evaluated for their efficacy using rigorous scientific studies. Detailed mechanism-based *in vitro* and *in vivo* studies should be performed for the characterization of their possible pharmacological effects. On the other hand, evaluation of possible toxicity of these medicinal plants/natural products is also important as these medicines are directly applied in eyes and can have not just potential benefits but also harmful effects. Although there are numerous studies going on at preclinical level, clinical evidence for efficacy is still the need of the hour.

## Author Contributions

DT, OS, AM, DG, HD, JE, and AA drafted the initial manuscript. All authors improved, contributed, and agreed on the final version of the manuscript.

## Conflict of Interest Statement

The authors declare that the research was conducted in the absence of any commercial or financial relationships that could be construed as a potential conflict of interest.

## Abbreviations

AGE: advanced glycation end products
AKR1B1, aldo-keto reductase family 1: member B1
AR: aldose reductase
ATP: adenosine triphosphate
BSA: bovine serum albumin
Cx: connexin
EPHA2: FRSA: free radical scavenging activity
GPX: glutathione peroxidase
GSH: glutathione
IL: interleukin
iNOS: inducible nitric oxide synthase
LPO: lipid peroxides
MAPKs: mitogen-activated protein kinase
NADPH: nicotinamide adenine dinucleotide phosphate
PKC: protein kinase C
RLAR: rat lens aldose reductase
RNS: reactive nitrogen species
ROS: reactive oxygen species
SOD: superoxide dismutase
TGF-β2: transforming growth factor $\beta_{2}$
TNF-α: tumor necrosis factor-α
UV: ultraviolet
VEGF: vascular endothelial growth factor
ZDF: zucker diabetic fatty.
